# Aquatic Macrophytes Are Associated With Variation in Biogeochemistry and Bacterial Assemblages of Mountain Lakes

**DOI:** 10.3389/fmicb.2021.777084

**Published:** 2022-01-27

**Authors:** Ella Ide DeWolf, William John Calder, Joshua Grant Harrison, Gregory Donald Randolph, Benjamin Edward Noren, Cynthia Weinig

**Affiliations:** ^1^Department of Botany, University of Wyoming, Laramie, WY, United States; ^2^Genome Technologies Laboratory, University of Wyoming, Laramie, WY, United States; ^3^CellDrop Biosciences Inc., Laramie, WY, United States

**Keywords:** water lilies, microbes, macrophytes, biogeochemistry, *Nuphar polysepalum*, mountain lakes

## Abstract

In aquatic systems, microbes likely play critical roles in biogeochemical cycling and ecosystem processes, but much remains to be learned regarding microbial biogeography and ecology. The microbial ecology of mountain lakes is particularly understudied. We hypothesized that microbial distribution among lakes is shaped, in part, by aquatic plant communities and the biogeochemistry of the lake. Specifically, we investigated the associations of yellow water lilies (*Nuphar polysepala)* with the biogeochemistry and microbial assemblages within mountain lakes at two scales: within a single lake and among lakes within a mountain range. We first compared the biogeochemistry of lakes without water lilies to those colonized to varying degrees by water lilies. Lakes with >10% of the surface occupied by water lilies had lower pH and higher dissolved organic carbon than those without water lilies and had a different microbial composition. Notably, cyanobacteria were negatively associated with water lily presence, a result consistent with the past observation that macrophytes outcompete phytoplankton and can suppress cyanobacterial and algal blooms. To examine the influence of macrophytes on microbial distribution within a lake, we characterized microbial assemblages present on abaxial and adaxial water lily leaf surfaces and in the water column. Microbial diversity and composition varied among all three habitats, with the highest diversity of microbes observed on the adaxial side of leaves. Overall, this study suggests that water lilies influence the biogeochemistry and microbiology of mountains lakes.

## Introduction

Bacteria play critical roles in mediating ecosystem processes in aquatic systems (Newton et al., 2011), including the cycling of biologically important nutrients (Clark et al., 2018). However, much remains to be studied regarding microbial ecology and biogeography, especially in high elevation lakes. Despite their underrepresentation in the literature, mountain lakes are important targets for study due to their sensitivity to ongoing environmental change (Catalan et al., 2006; Bartrons et al., 2012), importance for biogeochemical cycling (Brown et al., 2008), influence on downstream ecosystems (Alexander et al., 2007), and because they are a critical source of fresh water to humans and other animals (Viviroli et al., 2007).

A greater understanding of the factors influencing the distribution of microbes in mountain lakes is needed to clarify their contributions to ecosystem function (Green et al., 2008; Graham et al., 2016). For example, pH has been shown to be a major driver of microbial assemblages in terrestrial and aquatic ecosystems alike (Lindström et al., 2005, 2010; Bahram et al., 2018). Additionally, aquatic microbes may be affected by temperature (Lindström et al., 2005), the concentration and source of dissolved organic carbon (DOC) (Kritzberg et al., 2006), and nutrients such as nitrogen and phosphorus (Logue et al., 2012). However, the effects of these factors on microbial assemblages are not well defined, especially in mountain lakes.

Plant communities also play a role in structuring microbial assemblages. Such plant-microbe interactions have been well studied in terrestrial systems (Berendsen et al., 2012; Prober et al., 2015), and a growing body of literature indicates plants play a role in structuring the microbial composition of aquatic systems as well (Zeng et al., 2012; Srivastava et al., 2017). Aquatic plants, or macrophytes, influence aspects of water chemistry such as pH (Collins et al., 2004), dissolved oxygen (DO) (Netten et al., 2010; Vilas et al., 2017), and DOC (Mann and Wetzel, 1996). Macrophytes likely influence microbial composition in the water through these chemical changes, and the microbes themselves further shape the aquatic ecosystem and its biogeochemistry (Collins et al., 2004). Additionally, aquatic plants have been shown to uptake and store nutrients including phosphorus, nitrogen, and heavy metals and may outcompete algae and other phytoplankton for these nutrients (Takamura et al., 2003; Liu et al., 2016). Thus, aquatic vegetation plays a critical role in maintaining water quality and suppressing cyanobacterial and algal blooms in lakes and ponds (Dhote and Dixit, 2009; Bakker et al., 2010). In fact, researchers have proposed two alternative stable states with distinct microbial dynamics in freshwater ecosystems: a clear, macrophyte-dominated state, and a turbid phytoplankton-dominated state (Phillips et al., 2016; Wang Y. et al., 2020).

In addition to their effects on the chemical and microbial composition of surrounding water, which may influence microbial biogeography on a large scale (i.e., differences between lakes), macrophytes serve as hosts to microbial biofilms that form on the surfaces of leaves, stems, and roots (Pang et al., 2016; Srivastava et al., 2017), representing smaller scale influences on the spatial distribution of microbes within lakes (Souffreau et al., 2018). The composition of macrophyte-associated biofilms is distinct from that of the surrounding water (He et al., 2014, 2021) and is species-specific (Collins et al., 2004; Crump and Koch, 2008; Souffreau et al., 2018), indicating a complex relationship between the plant and the microbial assemblage. These microbial consortia may form a symbiotic association with the macrophyte in which the plant receives mineral nutrients and defensive immunity in return for organic carbon and oxygen (Srivastava et al., 2017) and may also contribute to nutrient cycling (Battin et al., 2003; Bartrons et al., 2012). However, macrophyte surfaces are heterogeneous environments and much remains to be learned about the spatial distribution and function of macrophyte-associated microbial assemblages (Schlechter et al., 2019).

The yellow water lily, *Nuphar polysepala*, (Nymphaeaceae) is native to western North America and can be found in shallow lakes and ponds throughout the Rocky Mountain region including the Snowy Range Mountains of Wyoming (Brewer and Smith, 1995). Water lilies are macrophytes that are rooted in sediment with leaves that extend to float on the surface of the water. The objective of this study was to examine how macrophytes, specifically *N. polysepala*, influence bacterial (hereinafter used interchangeably with “microbial”) distribution and biogeochemistry of mountain lakes at two scales: between different lakes and within a single lake. Our research questions are thus two-fold: (1) How does biogeochemistry and microbial composition differ between lakes with and without water lilies? And (2) how does microbial composition differ between the water column and water lily leaf surfaces? To address our first question, we characterized and compared biogeochemical measures and microbial assemblages in 12 mountain lakes, six of which were devoid of water lilies and six of which were colonized to varying degrees by water lilies. Additionally, to examine the influence of water lilies on the microbial biogeography within a lake, we characterized microbes present on different leaf surfaces of water lily plants and at different levels in the water column.

## Materials and Methods

### Sampling Design

To test for differences in biogeochemistry and microbial composition between lakes with and without water lilies, we collected samples from 12 mountain lakes in the Medicine Bow National Forest in southeast Wyoming (Table 1). Of these lakes, six were colonized by water lilies. Lakes ranged in elevation from 2,725 to 3,273 m and ranged in surface area from 2,300 to 113,000 m^2^. Lakes with water lilies varied in degree of lily growth from 0.9% surface coverage to 31.5% based on ImageJ analysis of satellite photos using the Color Segmentation plugin (Schindelin et al., 2012; Rueden et al., 2017). As this analysis may not be an accurate representation of water lily coverage at the time of sampling, we used it to classify lakes with water lilies into two groups based on surface coverage: few water lilies (<10% surface coverage) and many water lilies (>10% surface coverage). Water lilies generally begin to emerge as soon as the lakes thaw in the spring and flower within a month, but as the lakes sampled differed in elevation, the timing of water lily growth was different for each lake. To control for this difference, we sampled each lake when waterlilies were at peak flowering at that elevation, which took place between mid-July and mid-August of 2019. We selected lakes with few non-water lily plants, so that macrophyte-related effects might logically be attributed to the presence of water lilies and attempted to equalize other factors when selecting lakes; for instance, by selecting water lily and non-water lily lakes from across a similar elevation gradient.

**TABLE 1:** Details regarding the mountain lakes that were sampled.

Lake name	Water lily abundance	Elevation (m)	Longitude	Latitude	% Water lily surface coverage	Date sampled
Long Lake	Many	2725	41.5017	−106.3683	26.4%	7/17/19
Beaver Lake*	Many	2765	41.4914	−106.3447	31.5%	7/24/19
Hanging Lake	None	2771	41.3450	−106.1783	0%	7/24/19
Duck Lake*	None	2788	41.4817	−106.3664	0%	7/26/19
Slime Lake*	Few	2785	41.4828	−106.3672	0.8%	7/26/19
Frog Mt. Lake*	None	2875	41.4556	−106.3694	0%	7/29/19
Swamp lake*	Many	2878	41.4225	−106.4181	13.4%	7/30/19
Lily Lake	Few	3026	41.3511	−106.3872	8.2%	8/7/19
N. Banner Lake	None	3028	41.4150	−106.3583	0%	8/9/19
E. Banner Lake	Few	3069	41.4117	−106.3538	6.8%	8/9/19
Keystone Pond*	None	3118	41.3458	−106.3628	0%	8/22/19
Libby Lake	None	3273	41.3541	−106.2986	0%	8/22/19

*Names given by the researchers to previously unnamed lakes.

In each lake, eight sampling sites were chosen that were approximately evenly spaced in regions where water lilies were growing. In the case of lakes without waterlilies, we sampled where the depth was similar to sampled regions in water lily lakes (1–2 m deep). At each sampling site, we collected surface water in Nalgene bottles that had been bleached in a 10% bleach solution and autoclaved. Between 500–1,000 mL of water was filtered through a 0.2 μm filter and the filters were flash frozen and stored at −80°C for DNA extraction. Remaining water was stored at −20°C for biogeochemical analysis.

We performed additional sampling in Long Lake (see Table 1) to compare microbial composition on water lily leaf surfaces and in the water column. In addition to surface water collected as described above, we collected water 1–2 m deep (just above the sediment) using a Wildco beta water sampler (Wildco, Yulee, FA, United States). The sampler was cleaned in a 10% bleach solution for 30 min and rinsed in RO water prior to sampling. To remove additional microbes that may have been present before sampling, the first sample collected was discarded. As we were unable to clean the sampler in the field, we collected 5 L samples, opened the valve, and let the water flow for at least 5 s before collecting a 500 mL sample into a bleached and autoclaved Nalgene. Water samples were filtered and stored as described above.

Water lily leaf surfaces were swabbed with two separate sterile polyester-tipped swabs (Fischer Scientific, Pittsburg, PA, United States). From each plant, we sampled both adaxial (top) and abaxial (bottom) sides of both a fully submerged leaf and a leaf floating on the surface. Leaf swabs were transported on dry ice, flash frozen, and stored at −80°C for DNA extraction. We chose eight approximately evenly spaced sites around the lake and took one of each sample type (two water samples and four leaf samples) at each site.

### Biogeochemical Analysis

Temperature, pH, and DO concentrations were measured in the field using a YSI Professional Plus meter (Yellow Springs Instruments, Yellow Springs OH, United States). The field meter was not working properly at two lakes (Libby Lake and Keystone Pond) so we were not able to collect reliable data for these lakes, and they were removed from the biogeochemical analysis. For additional biogeochemical analysis, water was filtered through a 0.45 micron filter. DOC was measured on a Teledyne Tekmar Fusion TOC UV/persulfate analyzer and concentrations of each of the major cations (ammonium, calcium, lithium, magnesium, potassium, and sodium) and anions (bromide, chloride, fluoride, nitrate, nitrite, phosphate, and sulfate) were measured on a Thermo Scientific Dionex Integron HPIC according to the manufacturer’s instructions. We used the R packages lme4 (Bates et al., 2015) and lmerTest (Kuznetsova et al., 2017) to find biogeochemical measures associated with water lily coverage, while including lake as a random effect (R Core Team, 2020).

### Microbial Analysis

DNA was extracted from both filters and leaf swabs using a Qiagen PowerWater DNA extraction kit (Qiagen, Germantown, MD, United States) according to the manufacturer’s instructions. For leaf swabs, both swabs from each sample were combined in one extraction. The V4 region of the bacterial 16s rRNA gene was amplified from extracted eDNA using the 515F and 806R primers (Walters et al., 2016) at the University of Wyoming Genome Technologies Laboratory. Library preparations were performed according to a two-step PCR approach as follows:

Extracted DNAs were normalized to 10 ng/μl. Samples yielding less than 10 ng/μl were not concentrated. 3 μl KAPA HiFi HotStart PCR buffer, 0.3 μl KAPA HiFi HotStart PCR polymerase, 0.45 μl 10 M dNTPs, HPLC water, and 6 μl 0.25 μM barcoded primers were added to 2 μl of normalized DNA and PCR was performed using the following thermocycler conditions: 3 min at 95°C followed by 15 cycles of 30 s at 98°C, 30 s at 62°C, and 30 s at 72°C, with a final elongation for 5 min at 72°C. The purpose of this first PCR was to add unique molecular identifiers to each sample, amplify the target locus, and add a portion of the Illumina flowcell adaptor sequence. The resulting PCR products were cleaned using AxyPrep MagBead PCR Clean-Up kit (Axygen; Union City, CA, United States). A second PCR reaction was used to complete the addition of Illumina adaptors and further amplify template DNA. Thermal cycler conditions for this reaction were 3 min at 95°C followed by 19 cycles of 30 s at 98°C, 30 s at 55°C, and 30 s at 72°C, finishing with 5 min at 72°C. Samples were cleaned again with the AxyPrep MagBead PCR Clean-Up kit and pooled. The final library was sequenced at Psomagen labs (Rockville, MD, United States) on the Illumina NovaSeq 6000 platform using 2 × 250 paired-end sequencing.

Bioinformatics were performed using the DADA2 package in R (Callahan et al., 2016; R Core Team, 2020). Reads were filtered and trimmed based on quality, denoised, and merged, then chimeras removed and taxonomy assigned using the SILVA reference database (version 138) (Quast et al., 2013). Due to binned quality scores generated by the NovaSeq platform, the DADA2 algorithm likely does not resolve all sequencing errors. Therefore, we removed any amplicon sequence variants (ASVs) present in only one of the two technical replicates as well as ASVs with fewer than 10 total reads. These discarded ASVs represented only 0.4% of total reads. These bioinformatic methods recovered the expected taxa with no unexpected ASVs when tested on an equimolar DNA mock community of eight known taxa that was included on the sequencing lane with our samples (Zymo Research, CA, United States).

For each of our two questions, we used the following analyses to identify differences in microbial composition. We first tested for differences in alpha diversity using the R package Breakaway (Willis and Bunge, 2015; Willis et al., 2017) to model microbial richness on non-rarefied data and used the betta function to test for differences between groups of interest. We estimated Shannon diversity on rarefied sequence data using the R package phyloseq (McMurdie and Holmes, 2013) and used ANOVA models to test for differences in diversity between groups of interest. To identify course-grained differences in microbial composition, we used phyloseq and vegan packages (Oksanen et al., 2020) to create NMDS ordination plots and perform PERMANOVA analysis on Bray-Curtis dissimilarity matrices. Non-rarefied microbial count data was transformed to proportions for NMDS and PERMANOVA analyses. To identify environmental variables correlated with microbial composition from lakes with and without water lilies we used the *envfit* function in vegan to overlay biogeochemical measures onto the NMDS ordination (Oksanen et al., 2020).

We next identified taxa that were differentially abundant using a beta-binomial regression model in the R package corncob (Martin et al., 2020). We used family-level aggregated data in these models to reduce zeros in the data and look for more broad taxonomic patterns as many ASVs were specific to a certain lake or sample type. Rarefaction is not necessary when using beta-binomial models because relative abundances are the target of modeling. To identify taxa that are differentially abundant based on the presence of water lilies, we used water lily category (none, few, or many) as a covariate. Corncob was also used to identify taxa that were differentially abundant between sample types in Long Lake including the effect of both sample type (bottom of leaf, top of leaf, or water) and height in the water column on microbial abundance in the model design. We ran this model for each pair-wise comparison of sample types. For all corncob models, we used a false discovery rate cut-off of 0.01. All figures were made with ggplot2 (Wickham, 2016).

## Results

### Comparison of Lakes With and Without Water Lilies

**FIGURE 1:**
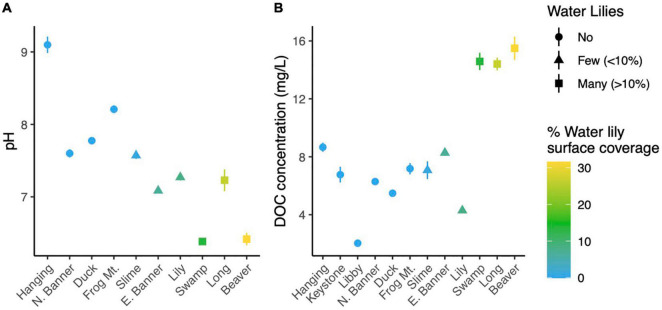
Average pH **(A)** and dissolved organic carbon **(B)** in each lake. Error bars (where visible) represent standard deviation.

To determine whether lakes with and without water lilies exhibited differences in biogeochemistry, we measured temperature, pH, DO, DOC, and major cations and anions, and used mixed effects models to identify measures that differed based on water lily abundance while considering the nested nature of our sampling design. The overall effect of water lilies was significant for pH and DOC (Figures 1A,B). Compared to lakes with no water lilies, pH was non-significantly lower by 0.86 units in lakes with few water lilies, and 1.49 units lower in lakes with many water lilies (*p* = 0.007). DOC was not significantly different in lakes with few water lilies compared to those without lilies, but DOC concentrations in lakes with many water lilies were more than double that of lakes without (beta = 8.76, *p* < 0.001). Sulfate and DO showed a similar pattern to pH (non-significantly lower in lakes with few water lilies and even lower in lakes with many water lilies) although the effect of many water lilies was only marginally significant (*p* = 0.047 and 0.036, respectively) and the overall effect of lilies was not significant. Aside from sulfate, none of the major cations or anions differed in concentration based on the presence of water lilies (Supplementary Figure 1).

To compare microbial composition in lakes with and without water lilies, we obtained 21,094,344 NovaSeq reads from 77 samples. Of these we retained 13,487,917 (63.9%) quality, non-chimeric reads that represented 6,686 bacteria AVSs. Eleven samples had read counts under 10,000 and were removed from analyses (leaving 66 samples). DNA concentrations from extracted samples ranged from 3.5 to 47.4 ng/μl with a mean of 20.3 ng/μl and did not differ based on water lily presence when the volume of lake water filtered was taken into account (Supplementary Figure 2). The most abundant phyla across all samples were Gammaproteobacteria (25.3% of total reads), Actinobacteria (21.5%), Verrucomicrobiota (20.5%), Bacteroidota (11.1%), and Cyanobacteria (7.8%) (Figure 4A).

**FIGURE 2:**
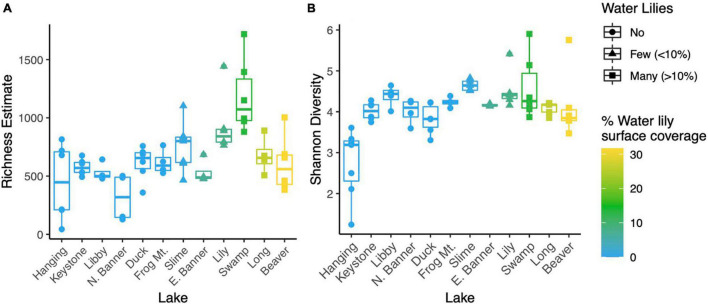
Point estimates for microbial richness estimated with Breakaway **(A)** and Shannon Diversity **(B)** for water samples from all 12 lakes.

**FIGURE 3:**
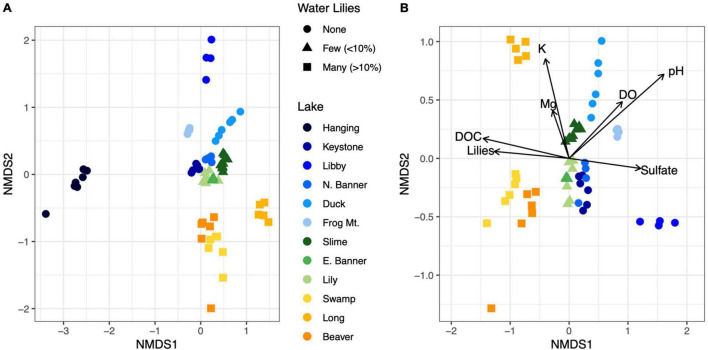
NMDS ordination of Bray-Curtis dissimilarities for microbial communities from all 12 lakes **(A)**. Biogeochemical parameters overlayed on an NMDS ordination of microbial communities for all lakes except Hanging Lake, a non-water lily lake with elevated pH and ion concentrations **(B)**. Biogeochemical measures significantly associated with the ordination (*p* < 0.01) are shown where the length of the arrow is proportional to the correlation coefficient (2r).

**FIGURE 4:**
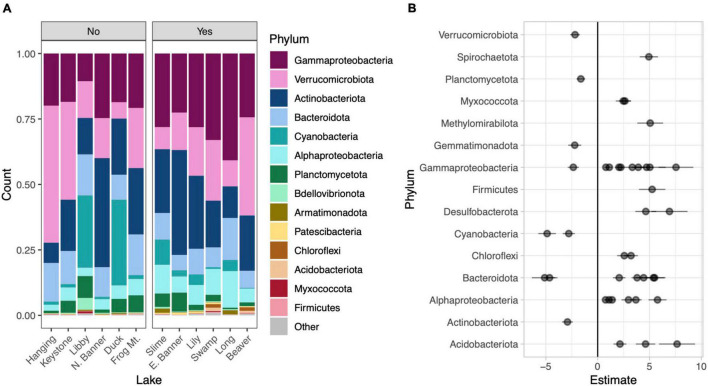
Phylum level taxonomic composition in each lake. Lakes are divided into those without water lilies (No) and with water lilies (Yes). Lakes with water lilies are ordered from left to right from lowest to highest water lily surface coverage. Only phyla with mean relative abundance greater than 0.01% across all samples are included and the phylum Proteobacteria has been broken down by subphylum **(A)**. Beta coefficient estimates from the corncob regression model for differentially abundant families that met our three criteria: (1) significantly differentially abundant between lakes with none and many water lilies, (2) the effect of few and many water lilies was the same direction, and (3) the magnitude of the effect of many water lilies was greater than the effect of few water lilies. Negative estimates indicate taxa more abundant in lakes with no water lilies while positive estimates indicate taxa more abundant in lakes with many water lilies. Individual families are represented by points and are grouped by phylum **(B)**.

Microbial richness was higher in lakes with water lilies versus those without water lilies, but was not higher in lakes with many (>10% surface coverage) water lilies as opposed to few (<10% surface coverage) (Figure 2A). Shannon diversity was not associated with water lily presence (Figure 2B). In terms of beta diversity, microbial composition differed based on water lily presence according to PERMANOVA analysis of Bray-Curtis dissimilarities (*p* < 0.001, *r*^2^ = 0.24, Figure 3A). Hanging Lake, a non-water lily lake, was much more basic, had elevated levels of most cations and anions, and had a different microbial composition than the other lakes. To ensure that Hanging Lake was not driving microbial differences between lakes with and without water lilies, we repeated this analysis omitting Hanging Lake and found that microbial composition still differed significantly based on the presence of water lilies (*p* < 0.001, *r*^2^ = 0.27). Six biogeochemical measures (pH, DOC, DO, sulfate, magnesium, and potassium) as well as percent water lily coverage were significantly correlated with the NMDS ordination both when Hanging Lake was included and when it was removed from the analysis (Figure 3B). Some variables, such as pH and DO were correlated, meaning it is not possible to separate out which if either might be causally associated with water lily presence.

Relative to lakes without water lilies, the corncob regression model revealed 77 bacterial families that were differentially abundant in lakes with water lilies. Of these, 60 were differentially abundant between lakes with no water lilies and lakes with many lilies and 27 were differentially abundant between lakes with no water lilies and few water lilies. We considered taxa that vary based on water lily coverage to be those that (1) were significantly differentially abundant between lakes with none and many water lilies and (2) the effect of few and many water lilies was in the same direction, and (3) the magnitude of the effect of many water lilies was greater than the effect of few water lilies. We identified 41 families that met these criteria and these are presented in Figure 4B and Supplementary Table 1.

### Comparison of Microsites Within Long Lake

**FIGURE 5:**
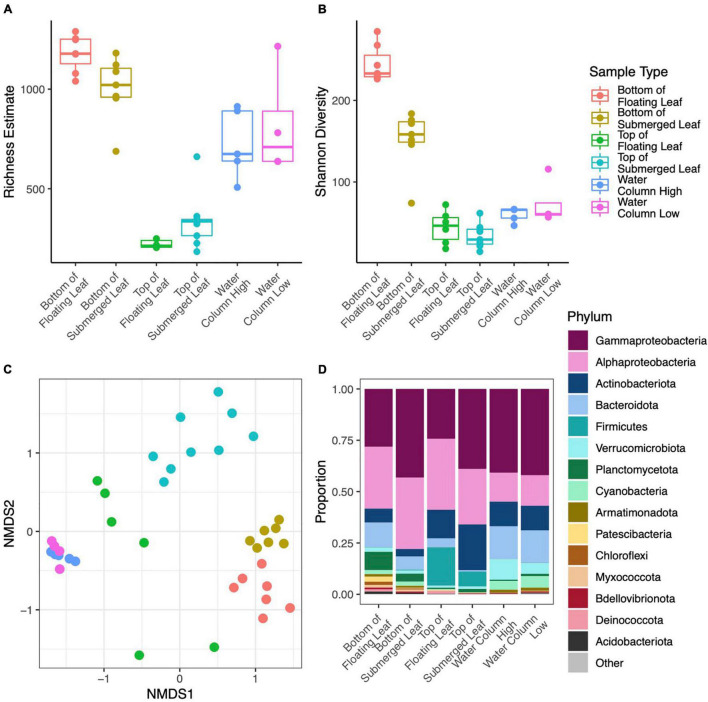
Point estimates for microbial richness estimated with Breakaway **(A)**, Shannon Diversity **(B)**, NMDS ordination of Bray-Curtis dissimilarities (colors correspond to the legend shown in the top row) **(C)**, and Phylum level taxonomic composition **(D)** for microbial samples collected from leaf surface and the water column within Long Lake. Panel **(D)** only includes phyla with mean relative abundance greater than 0.1% across all samples and the phylum Proteobacteria has been broken down by subphylum.

We obtained 10,219,819 raw reads from 44 samples from microsites within Long Lake. After processing, we retained 5,794,566 (56.7%) quality, non-chimeric, bacterial reads from 4155 ASVs. Many taxa were specific to a sample type with 1174 unique to the bottoms of leaves, 208 unique to the tops of leaves, and 916 unique to the water. We removed six samples with fewer than 10,000 reads. DNA concentrations of extracts ranged from 0.9 to 28.9 ng/μl and differed significantly (*p* < 0.001) between sample types with a mean of 10.3 for the bottoms of leaves, 2.3 for the tops of leaves, and 18.5 for water samples (Supplementary Figure 3). The dominant phyla on the bottoms of leaves were Gammaproteobacteria (35.0% of total reads), Alphaproteobacteria (32.0%), and Bacteroidota (9.5%), while those on top were Gammaproteobacteria (40.3%), Alphaproteobacteria (28.0%), Actinobacteria (19.4%), and those in the water were Gammaproteobacteria (40.9%), Alphaproteobacteria (14.2%), and Bacteroidota (16.0%) (Figure 5D).

In terms of alpha diversity, richness on the bottom side of leaves was over three times higher than the top of leaves (*p* < 0.001) and 42% higher than the water column (*p* = 0.012). Richness in the water column was over twice that of the tops of the leaves (*p* < 0.001) (Figure 5A). However, richness did not differ between samples from high and low in the water column, nor between sites in the lake. Similarly, Shannon diversity was 31% lower on the tops of leaves (*p* < 0.001) and 20% lower in the water column (*p* < 0.001) as compared to the bottoms of leaves. Shannon diversity was also 17% higher in the water as compared to the tops of the leaves (*p* = 0.002) (Figure 5B). Overall, Shannon diversity was slightly higher at the surface than lower in the water column (*p* = 0.04), and this difference was driven by leaf samples. PERMANOVA analysis showed that microbial composition differed significantly between sample types (*p* < 0.001, *r*^2^ = 0.500) and height in the water column (*p* = 0.002, *r*^2^ = 0.084), where the sample type explained more of the variation in microbial composition than height in the water column (Figure 5C). The site, or location within the lake, did not have a significant effect on microbial composition.

**FIGURE 6:**
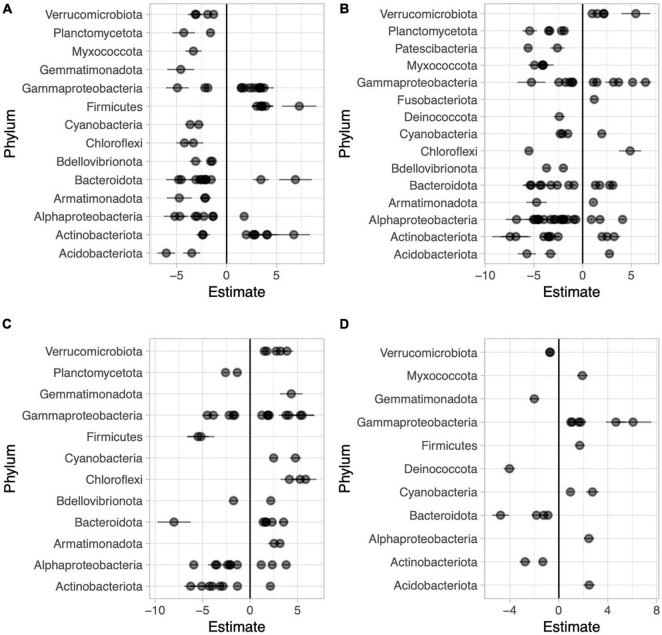
Beta coefficient estimates from the corncob regression model for differentially abundant families for each pairwise comparison between sample types in Long Lake: Abaxial (bottom) vs. adaxial (top) leaf surfaces, where negative beta estimates indicate taxa more abundant on the bottom while positive estimates indicate taxa more abundant top **(A)**, abaxial leaf surface vs. water, where negative beta estimates indicate taxa more abundant on the bottoms of leaves while positive estimates indicate taxa more abundant in the water **(B)**, adaxial leaf surface vs. water where negative beta estimates indicate taxa more abundant on the tops of leaves while positive estimates indicate taxa more abundant on adaxial surfaces **(C)**, and high vs. low in the water column across all samples where negative beta estimates indicate taxa more abundant on the surface and positive estimates indicate taxa more abundant lower in the water column **(D)**. Individual families are represented by points and are grouped by phylum.

We used the corncob beta-binomial regression model to identify families that were differentially abundant between sample types and performed this model for each pair-wise comparison of sample types. We identified 67 families that were differentially abundant between the two sides of the water lilies leaves out of 246 total families present in those sample types (Figure 6A and Supplementary Table 2). Comparing bottoms of leaves and the water column, we identified 85 (of 265) families that were differentially abundant (Figure 6B and Supplementary Table 3) and comparing tops of leaves and the water column, we identified 56 (of 261) families that were differentially abundant (Figure 6C and Supplementary Table 4). Additionally, across sample types, we found that 22 of the 273 total families were differentially abundant based on height (Figure 6D and Supplementary Table 5).

## Discussion

In this study, we uncovered strong associations between the presence of the macrophyte *N. polysepala* and variation in the biogeochemistry and microbial biogeography of mountain lakes at multiple spatial scales. First, we found that pH, and to a lesser extent, DO and sulfate, were lower in lakes with many water lilies and DOC was higher in lakes with many water lilies (>10% coverage) as compared to lakes without water lilies. None of these measures were significantly different between lakes with no water lilies and lakes with few water lilies (<10% coverage). The differences in pH and DO likely stem from respiration in under-water parts of the plant as well as microbial degradation of plant litter, processes that utilize oxygen and produce carbon dioxide (Collins et al., 2004). Macrophytes provide a significant portion of the organic carbon in aquatic ecosystems through photosynthesis and subsequent secretion and leaching of fixed carbon into the water, hence the elevated DOC we observed in lakes with water lilies (Mann and Wetzel, 1996).

Microbial composition also differed based on the presence of water lilies as shown on an NMDS ordination (Figure 3A). In particular, lakes with less than 10% water lily surface coverage had microbial compositions more similar to those of lakes with no water lilies, while lakes with greater than 10% water lily coverage had more distinct microbial composition. Together with our biogeochemical analysis, this indicates that water lilies can grow in a biogeochemical and microbial environment similar to lakes with no water lilies, and the changes we observe are more likely the result of water lily presence rather than the cause. However, further testing such as controlled greenhouse experiments are needed to test this hypothesis.

Several bacterial families were differentially abundant depending on water lily presence. While it is difficult to determine microbial function based purely on 16s-derived taxonomy, several of these differentially abundant taxa are worth noting. Two cyanobacterial families: Cyanobiaceae and Phormidiaceae were more abundant in lakes without water lilies, consistent with the theorized alternative states of shallow lakes: macrophyte dominated versus phytoplankton dominated states (Phillips et al., 2016). Additionally, members of the Phormidiaceae present in our samples are known to form cyanobacterial blooms and may produce toxins including anatoxins and microcystins (Salmaso et al., 2016; Bukowska et al., 2017). Thus, water lilies may play a role in reducing toxic cyanobacterial blooms. Two families of Desulfobacterota were also more abundant where water lilies were present. These taxa are known to reduce sulfate and degrade fatty acids and aromatic compounds as well as form syntrophic relationships with hydrogen metabolizing bacteria such as methanogens (Harmsen et al., 1998; Jackson et al., 1999). This may explain the slightly lower concentration of sulfates in these lakes. Finally, methanotrophs such as those in Methylomirabilaceae (Methylomirabilota) and Methylomonadaceae (Gammaproteobacteria) utilize compounds such as methane as a carbon source and thus may reduce methane emissions (Cui et al., 2015; Cabrol et al., 2020). This is important as wetlands are responsible for a substantial portion of global methane emissions (Chowdhury and Dick, 2013). When the biogeochemical parameters most associated with water lilies (pH and DOC) were statistically accounted for in the corncob model, fewer microbial taxa showed differential abundance associated with water lily presence. While experimental manipulations of water lilies are needed to disentangle the causality, the results support the hypothesis that water lilies may affect microbial biogeography *via* their effects on aquatic biogeochemistry.

Our second objective was to characterize the microbial assemblages on water lily leaf surfaces and analyze how they differ from those in the water column. Microbial diversity as measured by Shannon diversity and estimated richness, was much higher on the bottoms of leaves than the water column, supporting previous studies in which macrophyte-associated assemblages had higher richness and diversity than surrounding water (He et al., 2014, 2021). The increased diversity on the bottoms of leaves suggests that there are taxa found exclusively on the bottoms of leaves and not in the water. In fact, 1595 (55%) of the taxa identified on the bottoms of both floating and submerged leaves, were not found in any of the water samples. These taxa may be present at undetectable levels in the water column or may originate from another source such as sediment. Additionally, lakes with water lilies had higher microbial richness than lakes without. The diverse assemblage of microbes on the bottom surface of leaves may serve as a source for free-living or particle attached microbes in the water column, thus contributing to higher richness in the water column. Taxa more abundant on the leaf surface as opposed to water include potentially predatory bacteria from the phyla Myxococcota and Bdellovibrionota (Reichenbach, 1999; Sockett, 2009). The increased abundance of these taxa suggests that predation plays an important role in structuring the complex community present. Over one fourth of the families more abundant on the bottom of water lily leaves were Alphaproteobacteria, especially from the Rhizobiales order. While these bacteria exhibit diverse life styles and metabolism including purple sulfur bacteria, methanotrophs, and nitrogen fixers, many form symbiotic relationships with plants, as may be the case here (Sakarika et al., 2020; Wang S. et al., 2020). Finally, additional methanotrophic taxa (outside of Alphaproteobacteria) including Methylomonadaceae (mentioned above as more abundant in the water column where lilies are present) as well as two Gammaproteobacteria families were more abundant on leaf surfaces, again indicating that water lily-associated microbes may play a role in reducing wetland methane emissions.

While the underside of leaves hosted a more diverse microbial assemblage than the surrounding water, the microbes on the tops of leaves were less diverse and more variable than either the water or the bottoms of the leaves. DNA yields from the top surface were much lower than the bottom of leaves. DNA concentration has been used to approximate biomass or microbial abundance, and while this works best for samples with a defined quantity such as liters of water, milligrams of soil, or air sampled for a specified time, Gusareva et al. (2019), each side of the water lily leaf was swabbed with approximately the same effort and microbial abundance is thus likely much lower on the top as compared to the bottom. The difference in DNA yield was also reflected in read counts between sample types which may have influenced our alpha diversity calculations. Interestingly, this differential DNA yield, as well as differences in microbial composition between sides of the leaf, held true not only for floating leaves, in which the top and bottom are exposed to very different environments (air and water, respectively), but also for submerged leaves in which both sides of the leaf are exposed to a similar water environment. Additionally, submerged leaves are oriented more vertically and often curl around themselves, decreasing potential differences in UV radiation exposure between the tops and bottoms of submerged leaves. Thus, water lily leaf physiology, rather than the surrounding abiotic conditions, is likely cause of the differences we observed. Water lilies are hyperstomatous (they only have stomata on the adaxial side of the leaf), and the tops of leaves also contain a waxy cuticle that likely helps keep the leaf surface dry to promote efficient gas exchange for photosynthesis (Brewer and Smith, 1995). Our results indicate this cuticle may also prevent formation of a complex biofilm as seen on the bottoms of leaves. Additionally, the abaxial sides of Nymphaeaceae contain hydropotes, specialized gland-like cells, that are involved in the transport of nutrients and water in and out of the plant (Carpenter, 2006). These cells, or other secretion mechanisms may lead to the secretion of substances that promote the growth of the abaxial microbial biofilm.

We also identified several taxa that were differentially abundant between the two sides of the water lily leaves. Taxa more abundant on the tops of leaves include spore formers from both Actinobacteria and Firmicutes as well as taxa from Geodermatophilaceae (Actinobacteria) often found on rocks and known for their resistance to radiation and desiccation (Gtari et al., 2012). Meanwhile families more abundant on the bottoms of the leaves include taxa described above such as predatory Myxococcota and Bdellovibrionota, as well as potential nitrogen fixers and methanotrophs belonging to Proteobacteria. These results indicate that some microbes more abundant on the top of the leaves may be dormant or highly resistant cells originating from other sources that landed on the leaves while those on the bottom form a more complex community that plays an important role in biogeochemical cycling.

Our study demonstrates the associations of an aquatic macrophyte, *N. polysepala*, with the biogeochemistry and microbial biogeography of mountain lakes. We found that both biogeochemistry and microbial composition differed based on the presence of water lilies and that these biogeochemical and microbial differences are likely interconnected. We also characterized a diverse biofilm on the undersides of water lily leaves, which was much different from the microbial assemblage on top of leaves or in the water and may involve symbiotic associations with the plants. Our results suggest that an important ecological function of water lilies is maintaining higher microbial biodiversity and the microbial taxa associated water lilies likely perform important roles in biogeochemical cycling. Overall, this study demonstrates the complexity of interactions between microbes and macrophytes in understudied freshwater systems and suggests hypotheses for how these interactions shape lake biogeochemical profiles.

## Data Availability Statement

The datasets presented in this study can be found in online repositories. The names of the repository/repositories and accession number(s) can be found below: https://publications. pathfinder.arcc.uwyo.edu/Botany/Harrison/waterlily_ data.tar.gz, https://doi.org/10.15786/14555994.v1.

## Author Contributions

ED and CW designed the research. ED, WC, BN, and GR performed the research. ED, JH, and CW analyzed the data. All authors contributed to the writing of the manuscript and approved the submitted version.

## Conflict of Interest

BN was employed by the company CellDrop Biosciences Inc., Laramie. The remaining authors declare that the research was conducted in the absence of any commercial or financial relationships that could be construed as a potential conflict of interest.

## Publisher’s Note

All claims expressed in this article are solely those of the authors and do not necessarily represent those of their affiliated organizations, or those of the publisher, the editors and the reviewers. Any product that may be evaluated in this article, or claim that may be made by its manufacturer, is not guaranteed or endorsed by the publisher.
