# Supplementary material for: Aquatic Macrophytes Are Associated With Variation in Biogeochemistry and Bacterial Assemblages of Mountain Lakes
Source: Front Microbiol. 2022 Jan 27;12:777084. doi: 10.3389/fmicb.2021.777084 (PMC8828945; doi:10.3389/fmicb.2021.777084)
Supplement: Supplementary file 1 [file Data_Sheet_1.docx]

Supplementary Material

# Supplementary Figures

**Supplementary Figure 1:** Additional biogeochemical measures in each lake. Concentrations are shown in mg/L and temperature is shown in degrees Celsius. Error bars (where visible) represent standard deviation.

**Supplementary Figure 2:** DNA concentration in water samples by lake, adjusted for the amount of water filtered.

**Supplementary Figure 3:** Concentration of extracted DNA from microsites within long lake. All leave surfaces were sampled with two sterile swabs pooled into one extraction. 500ml was filtered for water samples.

# Supplementary Tables

**Supplementary Table 1:** Bacterial families that were differentially abundant by water lily presence based on three criteria: 1) significantly differentially abundant between lakes with none and many water lilies based on corncob regression model and 2) the effect of few and many water lilies was the same direction, and 3) the magnitude of the effect of many water lilies was greater than the effect of few water lilies. Negative beta estimates indicate taxa more abundant in lakes with no water lilies while positive beta estimates indicate taxa more abundant in lakes with many water lilies. Taxa are ordered from most to least abundant.

| Phylum | Class | Order | Family | Beta | Adjusted  p-value |
| --- | --- | --- | --- | --- | --- |
| Proteobacteria | Gammaproteobacteria | Burkholderiales | Burkholderiaceae | 0.79 | 3.99E-05 |
| Cyanobacteria | Cyanobacteriia | Synechococcales | Cyanobiaceae | -2.79 | 1.78E-04 |
| Proteobacteria | Gammaproteobacteria | Burkholderiales | Alcaligenaceae | 1.13 | 6.74E-04 |
| Proteobacteria | Alphaproteobacteria | Rickettsiales | Rickettsiaceae | 3.68 | 3.33E-07 |
| Planctomycetota | Planctomycetes | Gemmatales | Gemmataceae | -1.62 | 1.57E-03 |
| Actinobacteriota | Acidimicrobiia | Microtrichales | Ilumatobacteraceae | -2.91 | 1.23E-05 |
| Verrucomicrobiota | Verrucomicrobiae | Methylacidiphilales | Methylacidiphilaceae | -2.18 | 2.25E-04 |
| Proteobacteria | Alphaproteobacteria | Sphingomonadales | Sphingomonadaceae | 0.76 | 7.36E-04 |
| Proteobacteria | Alphaproteobacteria | Rhizobiales | Beijerinckiaceae | 1.10 | 9.41E-05 |
| Gemmatimonadota | Gemmatimonadetes | Gemmatimonadales | Gemmatimonadaceae | -2.21 | 4.41E-03 |
| Proteobacteria | Gammaproteobacteria | Methylococcales | Methylomonadaceae | 2.24 | 3.42E-07 |
| Proteobacteria | Gammaproteobacteria | Burkholderiales | SC-I-84 | 4.72 | 1.04E-07 |
| Bacteroidota | Bacteroidia | Sphingobacteriales | KD3-93 | -5.11 | 8.01E-04 |
| Proteobacteria | Alphaproteobacteria | Rhizobiales | Rhodomicrobiaceae | 5.77 | 3.42E-07 |
| Bacteroidota | Bacteroidia | Cytophagales | Cyclobacteriaceae | -4.64 | 1.15E-09 |
| Proteobacteria | Gammaproteobacteria | Burkholderiales | Chitinibacteraceae | -2.34 | 4.80E-04 |
| Myxococcota | Polyangia | Polyangiales | Phaselicystidaceae | 2.47 | 5.78E-03 |
| Proteobacteria | Alphaproteobacteria | Rickettsiales | SM2D12 | 1.39 | 5.03E-04 |
| Proteobacteria | Gammaproteobacteria | Burkholderiales | Ferrovaceae | 7.55 | 3.01E-04 |
| Bacteroidota | Bacteroidia | Chitinophagales | 37-13 | 3.80 | 1.25E-07 |
| Acidobacteriota | Vicinamibacteria | Vicinamibacterales | Vicinamibacteraceae | 4.62 | 1.05E-04 |
| Proteobacteria | Gammaproteobacteria | Steroidobacterales | Steroidobacteraceae | 3.92 | 2.77E-06 |
| Proteobacteria | Gammaproteobacteria | Burkholderiales | Chromobacteriaceae | 2.05 | 7.31E-05 |
| Bacteroidota | Bacteroidia | Bacteroidales | vadinHA17 | 4.44 | 1.23E-05 |
| Cyanobacteria | Cyanobacteriia | Cyanobacteriales | Phormidiaceae | -4.88 | 7.23E-06 |
| Spirochaetota | Spirochaetia | Spirochaetales | Spirochaetaceae | 4.94 | 1.18E-05 |
| Bacteroidota | Bacteroidia | Sphingobacteriales | LiUU-11-161 | 2.08 | 5.61E-03 |
| Acidobacteriota | Acidobacteriae | Acidobacteriales | Koribacteraceae | 7.66 | 3.85E-04 |
| Firmicutes | Negativicutes | Veillonellales-Selenomonadales | Selenomonadaceae | 5.25 | 8.20E-04 |
| Chloroflexi | Chloroflexia | Chloroflexales | Roseiflexaceae | 3.22 | 1.25E-04 |
| Bacteroidota | Bacteroidia | Sphingobacteriales | Lentimicrobiaceae | 5.49 | 6.38E-06 |
| Desulfobacterota | Syntrophobacteria | Syntrophobacterales | Syntrophobacteraceae | 6.93 | 1.20E-03 |
| Acidobacteriota | Acidobacteriae | Bryobacterales | Bryobacteraceae | 2.15 | 5.23E-03 |
| Methylomirabilota | Methylomirabilia | Methylomirabilales | Methylomirabilaceae | 5.07 | 9.38E-04 |
| Bacteroidota | Kryptonia | Kryptoniales | BSV26 | 5.38 | 2.38E-04 |
| Myxococcota | Polyangia | Haliangiales | Haliangiaceae | 2.63 | 3.61E-04 |
| Proteobacteria | Alphaproteobacteria | Rickettsiales | Fokiniaceae | 2.97 | 3.85E-04 |
| Desulfobacterota | Syntrophia | Syntrophales | Syntrophaceae | 4.64 | 2.38E-04 |
| Chloroflexi | Anaerolineae | Caldilineales | Caldilineaceae | 2.57 | 2.83E-03 |
| Proteobacteria | Gammaproteobacteria | Competibacterales | Competibacteraceae | 3.34 | 6.94E-04 |
| Proteobacteria | Gammaproteobacteria | Burkholderiales | Gallionellaceae | 5.03 | 7.99E-03 |

**Supplementary Table 2:** Bacterial families that were differentially abundant between abaxial and adaxial leaf surfaces based on corncob regression model. Negative beta estimates indicate taxa more abundant on abaxial surfaces while positive beta estimates indicate taxa more abundant on adaxial surfaces. Taxa are in order from most to least abundant.

| Phylum | Class | Order | Family | Beta | Adjusted  p-value |
| --- | --- | --- | --- | --- | --- |
| Proteobacteria | Gammaproteobacteria | Burkholderiales | Burkholderiaceae | 1.80 | 7.75E-08 |
| Actinobacteriota | Actinobacteria | Corynebacteriales | Mycobacteriaceae | 1.96 | 1.46E-06 |
| Proteobacteria | Gammaproteobacteria | Burkholderiales | Alcaligenaceae | 3.33 | 2.92E-07 |
| Proteobacteria | Gammaproteobacteria | Burkholderiales | Comamonadaceae | -1.88 | 1.03E-08 |
| Verrucomicrobiota | Verrucomicrobiae | Verrucomicrobiales | Verrucomicrobiaceae | -1.89 | 5.23E-04 |
| Planctomycetota | Planctomycetes | Gemmatales | Gemmataceae | -1.61 | 1.43E-03 |
| Proteobacteria | Alphaproteobacteria | Sphingomonadales | Sphingomonadaceae | -1.35 | 1.72E-03 |
| Actinobacteriota | Actinobacteria | Micrococcales | Microbacteriaceae | 2.63 | 3.19E-05 |
| Armatimonadota | Fimbriimonadia | Fimbriimonadales | Fimbriimonadaceae | -2.17 | 1.09E-04 |
| Proteobacteria | Alphaproteobacteria | Rhizobiales | Rhizobiales  Incertae Sedis | 1.76 | 1.86E-04 |
| Firmicutes | Bacilli | Bacillales | Bacillaceae | 3.55 | 9.75E-03 |
| Verrucomicrobiota | Verrucomicrobiae | Pedosphaerales | Pedosphaeraceae | -3.06 | 7.16E-04 |
| Proteobacteria | Alphaproteobacteria | Rhizobiales | Methylopilaceae | -3.11 | 5.03E-05 |
| Bacteroidota | Bacteroidia | Cytophagales | Microscillaceae | -2.52 | 4.67E-03 |
| Bacteroidota | Bacteroidia | Sphingobacteriales | env.OPS-17 | -2.05 | 1.73E-06 |
| Firmicutes | Bacilli | Lactobacillales | Lactobacillaceae | 3.58 | 8.79E-03 |
| Bacteroidota | Bacteroidia | Chitinophagales | Saprospiraceae | -2.70 | 1.03E-08 |
| Firmicutes | Bacilli | Staphylococcales | Staphylococcaceae | 3.35 | 1.80E-04 |
| Bacteroidota | Bacteroidia | Chitinophagales | Chitinophagaceae | -2.24 | 4.59E-06 |
| Proteobacteria | Alphaproteobacteria | Elsterales | Elsteraceae | -2.28 | 4.92E-05 |
| Proteobacteria | Gammaproteobacteria | Enterobacterales | Enterobacteriaceae | 3.74 | 3.42E-03 |
| Bacteroidota | Bacteroidia | Flavobacteriales | Flavobacteriaceae | -2.09 | 2.03E-04 |
| Proteobacteria | Gammaproteobacteria | Aeromonadales | Aeromonadaceae | 1.47 | 4.40E-03 |
| Firmicutes | Bacilli | Lactobacillales | Enterococcaceae | 3.91 | 2.56E-04 |
| Proteobacteria | Alphaproteobacteria | Caulobacterales | Hyphomonadaceae | -4.69 | 2.03E-04 |
| Cyanobacteria | Cyanobacteriia | Cyanobacteriales | Nostocaceae | -3.64 | 9.35E-06 |
| Acidobacteriota | Acidobacteriae | Bryobacterales | Bryobacteraceae | -6.04 | 1.07E-05 |
| Actinobacteriota | Actinobacteria | Kineosporiales | Kineosporiaceae | -2.39 | 5.29E-04 |
| Armatimonadota | Armatimonadia | Armatimonadales | Armatimonadaceae | -2.12 | 4.79E-03 |
| Proteobacteria | Gammaproteobacteria | Burkholderiales | Chitinibacteraceae | 2.70 | 2.95E-07 |
| Myxococcota | Polyangia | Polyangiales | Phaselicystidaceae | -3.32 | 2.37E-03 |
| Proteobacteria | Alphaproteobacteria | Rhizobiales | A0839 | -5.17 | 7.16E-04 |
| Bacteroidota | Bacteroidia | Chitinophagales | 37-13 | -3.08 | 8.65E-04 |
| Bacteroidota | Bacteroidia | Cytophagales | Spirosomaceae | -1.54 | 8.96E-03 |
| Proteobacteria | Gammaproteobacteria | Pseudomonadales | Pseudomonadaceae | 3.46 | 2.86E-04 |
| Actinobacteriota | Actinobacteria | Micrococcales | Cellulomonadaceae | -2.36 | 9.25E-03 |
| Proteobacteria | Alphaproteobacteria | Rhizobiales | Devosiaceae | -1.36 | 4.18E-03 |
| Bacteroidota | Bacteroidia | Sphingobacteriales | LiUU-11-161 | -4.80 | 3.45E-03 |
| Bdellovibrionota | Bdellovibrionia | Bacteriovoracales | Bacteriovoracaceae | -1.60 | 1.61E-04 |
| Proteobacteria | Gammaproteobacteria | Burkholderiales | Sulfuricellaceae | -4.92 | 1.48E-03 |
| Gemmatimonadota | Gemmatimonadetes | Gemmatimonadales | Gemmatimonadaceae | -4.58 | 8.96E-03 |
| Actinobacteriota | Actinobacteria | Propionibacteriales | Propionibacteriaceae | 2.84 | 5.93E-03 |
| Verrucomicrobiota | Verrucomicrobiae | Chthoniobacterales | Chthoniobacteraceae | -1.30 | 2.36E-03 |
| Bacteroidota | Bacteroidia | Flavobacteriales | NS9 marine group | -4.54 | 4.06E-07 |
| Bdellovibrionota | Bdellovibrionia | Bdellovibrionales | Bdellovibrionaceae | -1.43 | 3.42E-03 |
| Proteobacteria | Gammaproteobacteria | Legionellales | Legionellaceae | 2.34 | 1.53E-06 |
| Proteobacteria | Gammaproteobacteria | Methylococcales | Methylomonadaceae | -2.17 | 9.92E-06 |
| Cyanobacteria | Cyanobacteriia | Oxyphotobacteria  Incertae Sedis | Unknown Family | -2.78 | 9.70E-05 |
| Chloroflexi | Chloroflexia | Chloroflexales | Roseiflexaceae | -4.21 | 2.90E-04 |
| Armatimonadota | Chthonomonadetes | Chthonomonadales | Chthonomonadaceae | -4.73 | 4.05E-03 |
| Acidobacteriota | Acidobacteriae | Acidobacteriales | Acidobacteriaceae  Subgroup1 | -3.49 | 2.37E-03 |
| Proteobacteria | Gammaproteobacteria | Burkholderiales | SC-I-84 | 3.15 | 5.95E-03 |
| Bdellovibrionota | Oligoflexia | Silvanigrellales | Silvanigrellaceae | -3.10 | 3.88E-04 |
| Planctomycetota | Phycisphaerae | Tepidisphaerales | Tepidisphaeraceae | -4.28 | 3.64E-03 |
| Proteobacteria | Alphaproteobacteria | Micropepsales | Micropepsaceae | -2.93 | 3.42E-03 |
| Proteobacteria | Gammaproteobacteria | Burkholderiales | Chromobacteriaceae | 1.51 | 2.04E-03 |
| Firmicutes | Clostridia | Clostridiales | Clostridiaceae | 3.02 | 5.80E-05 |
| Chloroflexi | Anaerolineae | Caldilineales | Caldilineaceae | -3.31 | 9.16E-03 |
| Bacteroidota | Bacteroidia | Flavobacteriales | Weeksellaceae | 3.44 | 1.62E-03 |
| Verrucomicrobiota | Verrucomicrobiae | Opitutales | Opitutaceae | -3.12 | 2.19E-03 |
| Firmicutes | Clostridia | Lachnospirales | Lachnospiraceae | 7.27 | 1.81E-03 |
| Bacteroidota | Bacteroidia | Bacteroidales | Dysgonomonadaceae | 6.88 | 1.92E-03 |
| Proteobacteria | Gammaproteobacteria | Burkholderiales | T34 | 3.32 | 1.61E-03 |
| Actinobacteriota | Actinobacteria | Micrococcales | Micrococcaceae | 4.03 | 2.88E-03 |
| Actinobacteriota | Actinobacteria | Frankiales | Geodermatophilaceae | 6.72 | 2.04E-03 |
| Actinobacteriota | Actinobacteria | Micromonosporales | Micromonosporaceae | 2.81 | 3.45E-03 |
| Actinobacteriota | Actinobacteria | Corynebacteriales | Corynebacteriaceae | 4.05 | 5.95E-03 |

**Supplementary Table 3:** Bacterial families that were differentially abundant between abaxial leaf surfaces and the water column based on corncob regression model. Negative beta estimates indicate taxa more abundant on abaxial surfaces while positive beta estimates indicate taxa more abundant in the water column. Taxa are in order from most to least abundant.

| Phylum | Class | Order | Family | Beta | Adjusted  p-value |
| --- | --- | --- | --- | --- | --- |
| Proteobacteria | Gammaproteobacteria | Burkholderiales | Burkholderiaceae | 3.70 | 5.25E-15 |
| Actinobacteriota | Actinobacteria | Corynebacteriales | Mycobacteriaceae | 2.51 | 8.89E-10 |
| Proteobacteria | Gammaproteobacteria | Burkholderiales | Alcaligenaceae | 5.14 | 1.07E-11 |
| Proteobacteria | Gammaproteobacteria | Burkholderiales | Comamonadaceae | -1.33 | 9.32E-08 |
| Proteobacteria | Alphaproteobacteria | Rickettsiales | Rickettsiaceae | 4.11 | 9.19E-11 |
| Proteobacteria | Gammaproteobacteria | Burkholderiales | Methylophilaceae | -1.08 | 1.29E-03 |
| Bacteroidota | Bacteroidia | Flavobacteriales | Flavobacteriaceae | 1.76 | 1.22E-06 |
| Cyanobacteria | Cyanobacteriia | Synechococcales | Cyanobiaceae | 1.97 | 1.26E-03 |
| Bacteroidota | Bacteroidia | Cytophagales | Spirosomaceae | 1.31 | 6.04E-03 |
| Planctomycetota | Planctomycetes | Gemmatales | Gemmataceae | -3.50 | 6.60E-08 |
| Planctomycetota | Planctomycetes | Pirellulales | Pirellulaceae | -2.17 | 1.09E-06 |
| Proteobacteria | Alphaproteobacteria | Caulobacterales | Caulobacteraceae | 0.90 | 5.20E-06 |
| Proteobacteria | Alphaproteobacteria | Rickettsiales | Mitochondria | -2.31 | 2.28E-07 |
| Deinococcota | Deinococci | Deinococcales | Deinococcaceae | -2.40 | 2.10E-03 |
| Verrucomicrobiota | Verrucomicrobiae | Methylacidiphilales | Methylacidiphilaceae | 2.15 | 3.29E-07 |
| Verrucomicrobiota | Verrucomicrobiae | Verrucomicrobiales | Rubritaleaceae | 1.50 | 1.10E-04 |
| Verrucomicrobiota | Verrucomicrobiae | Chthoniobacterales | Terrimicrobiaceae | 2.20 | 2.06E-07 |
| Proteobacteria | Alphaproteobacteria | Sphingomonadales | Sphingomonadaceae | -1.89 | 6.60E-08 |
| Proteobacteria | Alphaproteobacteria | Rhizobiales | Beijerinckiaceae | -0.92 | 3.75E-04 |
| Armatimonadota | Fimbriimonadia | Fimbriimonadales | Fimbriimonadaceae | 1.10 | 5.17E-08 |
| Proteobacteria | Alphaproteobacteria | Acetobacterales | Acetobacteraceae | -0.70 | 4.00E-03 |
| Proteobacteria | Alphaproteobacteria | Rhodobacterales | Rhodobacteraceae | -1.53 | 5.00E-04 |
| Proteobacteria | Gammaproteobacteria | Legionellales | Legionellaceae | 1.47 | 8.12E-04 |
| Proteobacteria | Alphaproteobacteria | Rhizobiales | Rhizobiaceae | -2.94 | 5.36E-06 |
| Proteobacteria | Alphaproteobacteria | Rhizobiales | Pleomorphomonadaceae | -4.92 | 1.22E-07 |
| Bacteroidota | Bacteroidia | Sphingobacteriales | NS11-12  marine group | 2.77 | 3.17E-08 |
| Verrucomicrobiota | Verrucomicrobiae | Pedosphaerales | Pedosphaeraceae | 0.98 | 9.31E-04 |
| Proteobacteria | Alphaproteobacteria | Rhizobiales | Methylopilaceae | -6.80 | 6.50E-05 |
| Planctomycetota | Planctomycetes | Planctomycetales | Rubinisphaeraceae | -3.38 | 7.12E-09 |
| Bacteroidota | Bacteroidia | Cytophagales | Microscillaceae | -0.89 | 3.02E-03 |
| Patescibacteria | Saccharimonadia | Saccharimonadales | LWQ8 | -5.61 | 3.19E-10 |
| Actinobacteriota | Thermoleophilia | Solirubrobacterales | Solirubrobacteraceae | -2.52 | 4.80E-09 |
| Bacteroidota | Bacteroidia | Chitinophagales | Saprospiraceae | -1.42 | 1.13E-04 |
| Actinobacteriota | Actinobacteria | Frankiales | Sporichthyaceae | 2.00 | 9.56E-05 |
| Proteobacteria | Alphaproteobacteria | Rickettsiales | SM2D12 | -2.10 | 1.59E-08 |
| Bacteroidota | Bacteroidia | Flavobacteriales | Crocinitomicaceae | 3.10 | 6.68E-07 |
| Proteobacteria | Alphaproteobacteria | Elsterales | Elsteraceae | -4.57 | 2.28E-09 |
| Actinobacteriota | Acidimicrobiia | Microtrichales | Ilumatobacteraceae | -3.99 | 1.83E-11 |
| Chloroflexi | Anaerolineae | SBR1031 | A4b | -5.52 | 1.77E-10 |
| Myxococcota | Polyangia | Polyangiales | Polyangiaceae | -4.10 | 3.38E-05 |
| Proteobacteria | Gammaproteobacteria | Burkholderiales | T34 | 6.46 | 6.60E-08 |
| Proteobacteria | Gammaproteobacteria | Salinisphaerales | Solimonadaceae | -5.26 | 5.99E-03 |
| Actinobacteriota | Actinobacteria | Micrococcales | Intrasporangiaceae | -3.26 | 4.00E-03 |
| Proteobacteria | Alphaproteobacteria | Caulobacterales | Hyphomonadaceae | -4.47 | 3.29E-07 |
| Acidobacteriota | Acidobacteriae | Bryobacterales | Bryobacteraceae | -5.73 | 6.50E-05 |
| Actinobacteriota | Actinobacteria | Propionibacteriales | Nocardioidaceae | -3.48 | 3.95E-08 |
| Proteobacteria | Gammaproteobacteria | Methylococcales | Methylomonadaceae | -1.01 | 1.29E-03 |
| Actinobacteriota | Actinobacteria | Kineosporiales | Kineosporiaceae | -7.46 | 2.75E-03 |
| Myxococcota | Polyangia | Polyangiales | Phaselicystidaceae | -4.05 | 3.29E-07 |
| Proteobacteria | Alphaproteobacteria | Rhizobiales | A0839 | -2.82 | 1.07E-11 |
| Bacteroidota | Bacteroidia | Chitinophagales | 37-13 | -5.28 | 7.32E-05 |
| Proteobacteria | Gammaproteobacteria | Methylococcales | Methylococcaceae | 1.09 | 3.02E-03 |
| Actinobacteriota | Actinobacteria | Micrococcales | Cellulomonadaceae | -6.87 | 1.37E-03 |
| Bacteroidota | Bacteroidia | Sphingobacteriales | Sphingobacteriaceae | -2.61 | 6.73E-05 |
| Proteobacteria | Alphaproteobacteria | Rhizobiales | Devosiaceae | -4.69 | 3.20E-04 |
| Myxococcota | Myxococcia | Myxococcales | Myxococcaceae | -4.14 | 6.32E-03 |
| Proteobacteria | Alphaproteobacteria | Rhizobiales | Labraceae | -2.01 | 5.66E-04 |
| Proteobacteria | Gammaproteobacteria | Burkholderiales | Sulfuricellaceae | -1.76 | 1.03E-04 |
| Cyanobacteria | Vampirivibrionia | Obscuribacterales | Obscuribacteraceae | -2.31 | 9.59E-05 |
| Planctomycetota | Phycisphaerae | Tepidisphaerales | WD2101 soil group | -1.90 | 9.57E-04 |
| Bacteroidota | Bacteroidia | Cytophagales | Cyclobacteriaceae | -4.39 | 9.81E-07 |
| Actinobacteriota | Actinobacteria | Propionibacteriales | Propionibacteriaceae | -3.54 | 2.50E-03 |
| Proteobacteria | Alphaproteobacteria | Rhizobiales | Hyphomicrobiaceae | -3.29 | 1.87E-03 |
| Actinobacteriota | Acidimicrobiia | Microtrichales | Microtrichaceae | 3.23 | 5.02E-04 |
| Bacteroidota | Bacteroidia | Flavobacteriales | NS9 marine group | -5.38 | 2.15E-06 |
| Verrucomicrobiota | Omnitrophia | Omnitrophales | Omnitrophaceae | 5.47 | 5.68E-03 |
| Bdellovibrionota | Bdellovibrionia | Bdellovibrionales | Bdellovibrionaceae | -3.71 | 2.27E-09 |
| Acidobacteriota | Holophagae | Holophagales | Holophagaceae | 2.76 | 1.11E-05 |
| Cyanobacteria | Cyanobacteriia | Oxyphotobacteria  Incertae Sedis | Unknown Family | -2.07 | 2.06E-06 |
| Fusobacteriota | Fusobacteriia | Fusobacteriales | Fusobacteriaceae | 1.19 | 7.04E-03 |
| Chloroflexi | Chloroflexia | Thermomicrobiales | JG30-KF-CM45 | 4.89 | 1.76E-03 |
| Patescibacteria | Saccharimonadia | Saccharimonadales | Saccharimonadaceae | -2.60 | 5.86E-03 |
| Armatimonadota | Chthonomonadetes | Chthonomonadales | Chthonomonadaceae | -4.73 | 1.12E-03 |
| Proteobacteria | Alphaproteobacteria | Rhodospirillales | Magnetospirillaceae | -5.06 | 2.38E-05 |
| Cyanobacteria | Cyanobacteriia | Pseudanabaenales | Pseudanabaenaceae | -1.52 | 5.54E-06 |
| Bacteroidota | Bacteroidia | Sphingobacteriales | KD3-93 | -4.24 | 7.23E-03 |
| Bdellovibrionota | Oligoflexia | Silvanigrellales | Silvanigrellaceae | -1.98 | 4.81E-06 |
| Planctomycetota | Phycisphaerae | Tepidisphaerales | Tepidisphaeraceae | -5.45 | 8.15E-06 |
| Proteobacteria | Alphaproteobacteria | Micropepsales | Micropepsaceae | -3.97 | 2.78E-04 |
| Myxococcota | Polyangia | Haliangiales | Haliangiaceae | -4.95 | 2.61E-06 |
| Bacteroidota | Bacteroidia | Sphingobacteriales | AKYH767 | -3.26 | 8.12E-04 |
| Proteobacteria | Alphaproteobacteria | Holosporales | Holosporaceae | 1.78 | 2.90E-03 |
| Proteobacteria | Gammaproteobacteria | Competibacterales | Competibacteraceae | 3.17 | 5.86E-03 |
| Acidobacteriota | Acidobacteriae | Acidobacteriae | Acidobacteriae | -3.31 | 3.18E-04 |
| Proteobacteria | Gammaproteobacteria | Gammaproteobacteria  Incertae Sedis | Unknown Family | -2.43 | 8.77E-04 |

**Supplementary Table 4:** Bacterial families that were differentially abundant between adaxial leaf surfaces and the water column based on corncob regression model. Negative beta estimates indicate taxa more abundant on adaxial surfaces while positive beta estimates indicate taxa more abundant in the water column. Taxa are in order from most to least abundant.

| Phylum | Class | Order | Family | Beta | Adjusted  p-value |
| --- | --- | --- | --- | --- | --- |
| Proteobacteria | Gammaproteobacteria | Burkholderiales | Burkholderiaceae | 1.84 | 3.54E-08 |
| Proteobacteria | Gammaproteobacteria | Burkholderiales | Alcaligenaceae | 1.87 | 5.24E-06 |
| Cyanobacteria | Cyanobacteriia | Cyanobacteriales | Nostocaceae | 4.78 | 3.71E-06 |
| Proteobacteria | Alphaproteobacteria | Rickettsiales | Rickettsiaceae | 3.80 | 3.54E-08 |
| Bacteroidota | Bacteroidia | Flavobacteriales | Flavobacteriaceae | 3.53 | 1.81E-05 |
| Cyanobacteria | Cyanobacteriia | Synechococcales | Cyanobiaceae | 2.48 | 7.73E-05 |
| Bacteroidota | Bacteroidia | Cytophagales | Spirosomaceae | 1.72 | 1.79E-03 |
| Planctomycetota | Planctomycetes | Gemmatales | Gemmataceae | -1.35 | 9.24E-03 |
| Proteobacteria | Alphaproteobacteria | Caulobacterales | Caulobacteraceae | 2.34 | 8.18E-05 |
| Verrucomicrobiota | Verrucomicrobiae | Methylacidiphilales | Methylacidiphilaceae | 2.75 | 3.21E-07 |
| Proteobacteria | Gammaproteobacteria | Burkholderiales | Methylophilaceae | -1.78 | 4.95E-04 |
| Verrucomicrobiota | Verrucomicrobiae | Chthoniobacterales | Terrimicrobiaceae | 1.77 | 2.61E-06 |
| Bacteroidota | Bacteroidia | Chitinophagales | Chitinophagaceae | 2.33 | 2.47E-04 |
| Actinobacteriota | Actinobacteria | Micrococcales | Microbacteriaceae | -2.86 | 4.89E-04 |
| Armatimonadota | Fimbriimonadia | Fimbriimonadales | Fimbriimonadaceae | 3.15 | 7.72E-07 |
| Proteobacteria | Alphaproteobacteria | Rhizobiales | Rhizobiales  Incertae Sedis | -2.11 | 1.59E-06 |
| Verrucomicrobiota | Verrucomicrobiae | Opitutales | Opitutaceae | 3.19 | 3.52E-03 |
| Verrucomicrobiota | Verrucomicrobiae | Pedosphaerales | Pedosphaeraceae | 3.88 | 1.65E-04 |
| Planctomycetota | Planctomycetes | Planctomycetales | Rubinisphaeraceae | -2.59 | 9.51E-08 |
| Bacteroidota | Bacteroidia | Cytophagales | Microscillaceae | 1.62 | 3.25E-03 |
| Proteobacteria | Alphaproteobacteria | Rhodobacterales | Rhodobacteraceae | -1.35 | 1.52E-04 |
| Proteobacteria | Alphaproteobacteria | Rhizobiales | Pleomorphomonadaceae | -5.93 | 6.56E-09 |
| Bacteroidota | Bacteroidia | Chitinophagales | Saprospiraceae | 1.37 | 2.49E-03 |
| Actinobacteriota | Actinobacteria | Frankiales | Sporichthyaceae | 2.13 | 3.41E-06 |
| Firmicutes | Bacilli | Staphylococcales | Staphylococcaceae | -5.18 | 8.06E-03 |
| Proteobacteria | Alphaproteobacteria | Rickettsiales | SM2D12 | -2.38 | 9.91E-07 |
| Proteobacteria | Alphaproteobacteria | Elsterales | Elsteraceae | -2.09 | 6.81E-03 |
| Proteobacteria | Gammaproteobacteria | Aeromonadales | Aeromonadaceae | -1.63 | 1.91E-03 |
| Actinobacteriota | Thermoleophilia | Solirubrobacterales | Solirubrobacteraceae | -1.34 | 1.52E-04 |
| Proteobacteria | Gammaproteobacteria | Burkholderiales | T34 | 3.81 | 5.24E-06 |
| Verrucomicrobiota | Verrucomicrobiae | Verrucomicrobiales | Verrucomicrobiaceae | 1.50 | 4.85E-03 |
| Firmicutes | Bacilli | Lactobacillales | Enterococcaceae | -5.49 | 1.11E-03 |
| Armatimonadota | Armatimonadia | Armatimonadales | Armatimonadaceae | 2.52 | 5.00E-03 |
| Actinobacteriota | Actinobacteria | Propionibacteriales | Nocardioidaceae | -3.17 | 3.93E-06 |
| Proteobacteria | Gammaproteobacteria | Methylococcales | Methylomonadaceae | 1.23 | 2.58E-03 |
| Proteobacteria | Gammaproteobacteria | Burkholderiales | Chitinibacteraceae | -2.20 | 3.10E-06 |
| Proteobacteria | Alphaproteobacteria | Paracaedibacterales | Paracaedibacteraceae | 1.17 | 1.60E-03 |
| Proteobacteria | Gammaproteobacteria | Methylococcales | Methylococcaceae | 2.01 | 9.44E-05 |
| Proteobacteria | Gammaproteobacteria | Pseudomonadales | Pseudomonadaceae | -4.50 | 3.71E-06 |
| Bdellovibrionota | Bdellovibrionia | Bacteriovoracales | Bacteriovoracaceae | 2.18 | 3.85E-06 |
| Chloroflexi | Chloroflexia | Chloroflexales | Roseiflexaceae | 4.14 | 2.03E-03 |
| Proteobacteria | Gammaproteobacteria | Burkholderiales | Sulfuricellaceae | 4.08 | 5.00E-03 |
| Actinobacteriota | Actinobacteria | Propionibacteriales | Propionibacteriaceae | -6.26 | 8.45E-07 |
| Proteobacteria | Alphaproteobacteria | Rhizobiales | Rhizobiaceae | -3.65 | 7.72E-07 |
| Proteobacteria | Gammaproteobacteria | Burkholderiales | B1-7BS | 5.30 | 8.52E-03 |
| Proteobacteria | Gammaproteobacteria | Steroidobacterales | Steroidobacteraceae | 5.48 | 2.52E-03 |
| Gemmatimonadota | Gemmatimonadetes | Gemmatimonadales | Gemmatimonadaceae | 4.34 | 8.06E-03 |
| Chloroflexi | Anaerolineae | Anaerolineales | Anaerolineaceae | 5.27 | 1.52E-04 |
| Proteobacteria | Gammaproteobacteria | Burkholderiales | Chromobacteriaceae | -3.88 | 1.52E-04 |
| Chloroflexi | Anaerolineae | Caldilineales | Caldilineaceae | 5.85 | 4.98E-04 |
| Bacteroidota | Bacteroidia | Flavobacteriales | Weeksellaceae | -8.01 | 1.60E-03 |
| Proteobacteria | Alphaproteobacteria | Rhizobiales | Xanthobacteraceae | -3.50 | 5.80E-03 |
| Bdellovibrionota | Bdellovibrionia | Bdellovibrionales | Bdellovibrionaceae | -1.74 | 8.06E-03 |
| Actinobacteriota | Actinobacteria | Frankiales | Frankiaceae | -5.10 | 4.53E-03 |
| Actinobacteriota | Actinobacteria | Micromonosporales | Micromonosporaceae | -4.00 | 9.34E-03 |
| Actinobacteriota | Actinobacteria | Corynebacteriales | Corynebacteriaceae | -4.28 | 4.93E-03 |

**Supplementary Table 5:** Bacterial families from leaf surfaces and water column that were differentially abundant based on height in the water column in the corncob regression model. Negative beta estimates indicate taxa more abundant on the surface while positive beta estimates indicate taxa more abundant lower in the water column. Taxa are in order from most to least abundant.

| Phylum | Class | Order | Family | Beta | Adjusted  p-value |
| --- | --- | --- | --- | --- | --- |
| Proteobacteria | Gammaproteobacteria | Burkholderiales | Methylophilaceae | 1.09 | 3.94E-04 |
| Verrucomicrobiota | Verrucomicrobiae | Verrucomicrobiales | Verrucomicrobiaceae | -0.70 | 5.41E-06 |
| Deinococcota | Deinococci | Deinococcales | Deinococcaceae | -4.03 | 2.37E-07 |
| Verrucomicrobiota | Verrucomicrobiae | Chthoniobacterales | Terrimicrobiaceae | -0.75 | 7.49E-05 |
| Bacteroidota | Bacteroidia | Chitinophagales | Chitinophagaceae | -0.89 | 1.92E-07 |
| Bacteroidota | Bacteroidia | Cytophagales | Hymenobacteraceae | -4.76 | 5.41E-06 |
| Proteobacteria | Gammaproteobacteria | Burkholderiales | SC-I-84 | 4.66 | 1.16E-04 |
| Proteobacteria | Gammaproteobacteria | Salinisphaerales | Solimonadaceae | 1.62 | 7.27E-03 |
| Actinobacteriota | Actinobacteria | Micrococcales | Intrasporangiaceae | -2.76 | 5.25E-05 |
| Proteobacteria | Gammaproteobacteria | Methylococcales | Methylomonadaceae | 0.98 | 3.46E-04 |
| Actinobacteriota | Actinobacteria | Kineosporiales | Kineosporiaceae | -1.31 | 1.18E-03 |
| Gemmatimonadota | Gemmatimonadetes | Gemmatimonadales | Gemmatimonadaceae | -2.00 | 4.80E-04 |
| Myxococcota | Myxococcia | Myxococcales | Anaeromyxobacteraceae | 1.92 | 2.49E-03 |
| Proteobacteria | Gammaproteobacteria | Burkholderiales | B1-7BS | 6.06 | 3.68E-03 |
| Bacteroidota | Bacteroidia | Flavobacteriales | NS9 marine group | -1.23 | 2.47E-03 |
| Proteobacteria | Gammaproteobacteria | Burkholderiales | Chitinimonadaceae | 1.79 | 6.03E-03 |
| Acidobacteriota | Holophagae | Holophagales | Holophagaceae | 2.48 | 3.06E-04 |
| Proteobacteria | Alphaproteobacteria | Caedibacterales | Caedibacteraceae | 2.45 | 1.07E-04 |
| Cyanobacteria | Cyanobacteriia | Pseudanabaenales | Pseudanabaenaceae | 0.95 | 4.80E-04 |
| Bacteroidota | Bacteroidia | Sphingobacteriales | AKYH767 | -1.82 | 1.18E-03 |
| Firmicutes | Bacilli | Mycoplasmatales | Mycoplasmataceae | 1.71 | 3.68E-03 |
| Cyanobacteria | Cyanobacteriia | Cyanobacteriales | Phormidiaceae | 2.75 | 3.06E-04 |
